# The Impact of the Myeloid Response to Radiation Therapy

**DOI:** 10.1155/2013/281958

**Published:** 2013-04-07

**Authors:** Michael J. Gough, Kristina Young, Marka Crittenden

**Affiliations:** ^1^Earle A. Chiles Research Institute, Robert W. Franz Cancer Center, Providence Portland Medical Center, 4805 NE Glisan, Portland, OR 97213, USA; ^2^The Department of Radiation Medicine, Oregon Health & Science University, Portland, OR 97239, USA; ^3^The Oregon Clinic, Portland, OR 97213, USA

## Abstract

Radiation therapy is showing potential as a partner for immunotherapies in preclinical cancer models and early clinical studies. As has been discussed elsewhere, radiation provides debulking, antigen and adjuvant release, and inflammatory targeting of effector cells to the treatment site, thereby assisting multiple critical checkpoints in antitumor adaptive immunity. Adaptive immunity is terminated by inflammatory resolution, an active process which ensures that inflammatory damage is repaired and tissue function is restored. We discuss how radiation therapy similarly triggers inflammation followed by repair, the consequences to adaptive immune responses in the treatment site, and how the myeloid response to radiation may impact immunotherapies designed to improve control of residual cancer cells.

## 1. Introduction

Radiation therapy is the most efficient system to deliver site-specific cytotoxicity in patients. The dominant focus of radiation therapy research for the past four decades has been extending the therapeutic margin of radiation therapy by increasing the radiosensitivity of cancer cells with radiosensitizing drugs, or decreasing the sensitivity of normal cells with radioprotectants. Despite this effort, such agents have shown limited clinical impact. Instead, advanced treatment planning and delivery techniques have permitted a dramatic escalation in the dose that can be safely delivered to a target site while sparing surrounding tissues. The imaging, physics, technology, and clinical science capability supporting these techniques have extended the use of radiation therapy such that it is now an alternative to surgery to control multiple individually targeted metastatic lesions in patients. The limited contribution of radiosensitizers and radioprotectors to clinical radiation therapy may relate to the fact that a significant portion of the tumor is normal tissue. For example, cancer cells subvert the conventional physiologic process of angiogenesis and vasculogenesis, orchestrated by myeloid cells, fibroblasts, smooth muscle cells, and endothelial cells. This stromal component of tumors can be highly relevant to outcome in cancer patients [1]. While the stromal cells may be abnormally manipulated by cancer cells, none of the stromal cells are transformed and may be critical targets within the treatment field [2]. The limited capacity of the stromal cells to keep up with the constant demands of cancer cell expansion results in hypoxia, which, despite escalating radiation doses, remains the single largest obstacle to efficacy in radiation therapy [3]. Following radiation-mediated death of cancer cells, the tumor can remain for a prolonged period, resulting in evolution of the site into scar tissue [4, 5], repopulation with residual cancer cells [6], or a slow dissolution. This interaction between the cancer cells and the stromal cells of the tumor represents a novel frontier in radiation research, particularly in view of the increased understanding of the immune biology of cancer. 

We believe that the tumor macrophage lies at the center of the normal tissue response to radiation therapy. Macrophages are manipulated by cancer cells to drive angiogenesis, invasion, and metastases and establish an immune environment that limits control of antigenic cancer cells by adaptive immunity. When cancer cells are killed by radiation, macrophages are the primary tumor-resident population of phagocytes, and their exposure to dying cells influences the immune balance of the treated tumor. Finally, macrophages are a central cell directing wound healing, and the repair response of macrophages in irradiated tissues influences the transition to fibrosis and may become increasingly relevant with the expansion in hypofractionated radiation therapies. This review considers the response of tumor macrophages following radiation therapy, their contribution to the success and failure of treatment, and our ability to target the macrophage response to influence the outcome of radiation therapy.

## 2. Radiosensitization, Radioprotection, and the Repair Response

An array of proteins and signaling pathways regulate the *sensitivity* of cells to programmed cell death pathways triggered by the DNA damage. Over the years, an equivalent array of strategies aimed to regulate these pathways has been studied. A frequent “hallmark” of cancer cells is decreased sensitivity to apoptotic signaling [7], for example, through overexpression of antiapoptotic genes such as Bcl2. Therapies interrupting these protective pathways (reviewed in [8]) show early promise in combination with cytotoxic therapies [9]. Interestingly, such therapies can have unintended consequences. While modulating apoptotic sensitivity can increase the sensitivity of cells receiving potentially lethal radiation doses, carcinoma cells receiving a toxic dose of radiation do not become viable, clonogenic cells where apoptosis is blocked. Instead, cell death may occur through a distinct mechanism. Thus, in cancer cells expressing low levels of Bcl2, cytotoxic therapy inducing DNA damage may cause death through apoptosis. In cells expressing high levels of Bcl2, death still occurs, but through nonapoptotic pathways [10]. Apoptosis is known to be immunosuppressive and anti-inflammatory, and results in efficient phagocytosis and clearance of dying cells and antigens [11]. By contrast, non-apoptotic death, which may be associated with high Bcl2 levels in the target cell [10], results in release of immunological adjuvants and is immunogenic and inflammatory, resulting in improved tumor control by endogenous immune mechanisms [10, 12, 13]. While reducing clonogenicity is the principle aim in radiation therapy, this unintended consequence of cell death on immune responses may be missed in models using xenograft cells and tissues in immune-incompetent animals [9], and these factors may contribute to the difficulty of clinical translation of many experimental approaches [14]. The immune system is a critical contributor to the tumor environment [15, 16], and adaptive immune function appears to be an important contributor to the efficacy of radiation therapy [17, 18]. Thus, enhanced apoptosis in the tumor following radiation therapy may be counterproductive. Data exists to support this proposal; repopulation of tumors with cancer cells following radiation therapy is an important cause of treatment failure [6], and cancer cells undergoing programmed apoptosis have been shown to accelerate repopulation by residual viable cells in part through arachidonic acid cascade and PGE_2_ formation [19]. In murine models, cancer cells engineered to lack the apoptotic trigger molecule Caspase 3 were more effectively treated with radiation therapy, and patients lacking caspase 3 showed a significantly better outcome than patients with caspase 3 (and, hence, with a functional apoptotic response) [19]. These data present a case that radiation sensitivity should not be considered in isolation. The sensitivity and mode of death of a cancer cell will depend on many factors, but in the event that a cancer cell dies, the consequence of that death to the surrounding cells may be extremely influential to outcome [20].

Conventionally fractionated radiation therapy has traditionally been considered immunosuppressive [21]. This is due, in part, to the early apoptotic death occurring in lymphocytes following low doses of radiation [22]. However, lymphocyte subsets have distinct radiosensitivities, with immunosuppressive T regulatory cells being more radioresistant [23]. In addition, macrophages are relatively radioresistent, and their survival coupled with recruitment results in increased proportions of macrophages in the tumor stroma following radiation [24–26]. In contrast to conventionally fractionated radiation, hypofractionated radiation therapy with large doses per fraction has unique radiobiologic features contributing to distinct immunobiology. Doses greater than approximately 10 Gy per fraction lead to endothelial cell membrane damage and activation of the ceramide pathway triggering apoptosis via acid sphingomyelinase [27] and is a major cause of radiation tissue damage at these doses [28]. Such lipid damage, which is more prominent at higher doses of radiation, can activate the SAPK/JNK pathway upregulating NF*κ*B and subsequent expression of MHC, cytokines, and inflammatory mediators [29, 30]. Thus, increased MHC expression is dose dependent, and most profound following SBRT doses [29]. Further, high dose radiation leads to damage-associated molecular patterns (DAMPs) that trigger expression and release of cytokines, chemokines, and inflammatory mediators [30]. The potential for increased vascular permeability resulting from endothelial apoptosis in combination with DAMP-triggered cytokine release and upregulation of MHC and costimulatory molecules can create a proinflammatory environment in the irradiated tumor. The therapeutic efficacy of SBRT may require this pro-inflammatory environment to generate an increased adaptive immune contribution following treatment, resulting greater CD8 T-cell priming in draining lymph nodes following high dose radiation [17] and a dependence on these immune cells for full efficacy [17, 18].

While tumors have been described as “wounds that do not heal” [31], the tumor is a wound that is continuously *attempting* to heal. The features of wound healing are a general indicator of advanced cancer and poor response to treatment [32–34], and this inflammatory pattern of wound healing extends beyond the malignant cells and into surrounding tissue where it can also be a prognostic factor [32]. A gene expression profile that provides a “wound-response signature” was predictive of local recurrence following breast-conserving surgery in breast cancer patients [35]. Interestingly, in patients receiving surgical therapies, the normal process of wound healing in the postoperative period has been linked to outgrowth of residual cancer cells [36]. Thus, while a fibrotic pancreas may be considered a good predictor for healing of anastomosis [37], a high level of wound-phenotype macrophages infiltrating pancreatic tumors is a marker of poor prognosis, with lower overall survival [38]. That surgical treatments initiate wound healing is easily understood, and healing of surgical wounds can be perturbed by radiation therapy [39]. However, in response to the damage that they cause, both chemotherapy and radiation therapy directly initiate something closely analogous to a classical wound healing response.

The wound healing response to radiation therapy is evidenced by the problem of pulmonary fibrosis in lung cancer patients. Up to 15% of patients receiving high dose radiation for therapy of lung cancer exhibit pneumonitis (reviewed in [40]), but subsequent lung fibrosis has been shown to occur at a much higher frequency where patients are prospectively screened [5]. This inflammatory pneumonitis is also a feature of chemotherapies [40], and while the acute chemotherapy pneumonitis is mostly seen to resolve once the chemotherapy is halted, at late stages patients can develop pulmonary fibrosis and restrictions in lung function [41]. Radiation-induced pneumonitis has an acute phase ranging from 2–6 months following completion of therapy [4], and the likelihood of lung injury is linked to the dose and the volume of lung irradiated. The chronic aspect of pneumonitis develops over 6–24 months and can be a source of significant morbidity. Normal wound healing responses transition from local inflammation to proliferation and remodeling [39]. The pathology in pulmonary fibrosis is driven by incomplete resolution of inflammation resulting in progressive fibrosis—leading to loss of organ function [42]. Macrophages are critical in the transition points between damage and repair but play distinct roles in the early versus late stages of resolution. Depletion of macrophages during the initial inflammatory insult prevents fibrosis from developing [43]. Depletion of macrophages at late stages, where remodeling and resolution is taking place, results in persistent fibrosis [43]. These data are closely analogous to those during healing of a skin wound; depletion of macrophages early following injury reduced scar formation and improved healing, while removal during resolution dramatically reduced healing [44]. In these models, macrophages are playing dual roles where they participate in M1-type responses early in inflammation and convert to proresolution M2 responses at later stages. A series of cytokines sustains the acute phase of radiation-induced inflammation [45], and these cytokine patterns match the status of inflammatory macrophage differentiation in the site of radiation [46]. The initiating proinflammatory cascade has been linked to production of cytokines, including the M1 cytokine TNF*α* [47, 48]. Later in this inflammatory cascade, the M2 cytokine TGF*β* is expressed [49, 50], and blocking TGF*β* function *in vivo* has been shown to diminish pneumonitis and functional impairment in animal models [51–53]. Variations in TGF*β* alleles have in some studies been associated with a genetic risk of radiation toxicity [54]. This M2 fibrotic pattern holds true in other cases of pathological fibrosis, such as the development of liver or pancreatic fibrosis by chronic inflammation, which is also driven to a large degree by the cytokine TGF*β* [55, 56]. 

**Figure 1:**
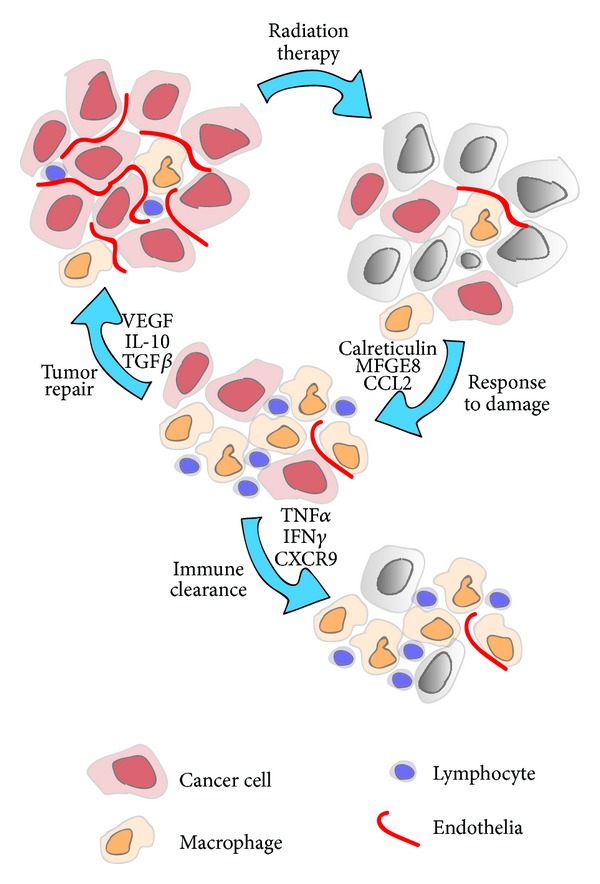
Tumor destruction or repair following cytotoxic therapy. High dose radiation therapy of tumors results in death of cancer cells, endothelial cells, and lymphocytes, but small numbers of cancer cells with clonogenic potential can survive. Cancer cell death triggers phagocytic receptors on radioresistant tumor macrophages and results in recruitment of both lymphocytes and macrophages to the treatment site. The immune response and the inflammatory milieu at the treatment site may influence outcome; a proinflammatory environment can permit immune-mediated clearance of residual cancer cells, while an anti-inflammatory environment can suppress adaptive immunity and repair the tumor environment for cancer recurrence.

In classical infectious models of acute inflammation and resolution, neutrophil death and their phagocytosis by macrophages is a critical initiating event for inflammatory resolution [57, 58]. Sterile inflammation similarly induces a locally destructive phase accompanied by neutrophils, which upon completion results in neutrophil death and removal by a secondary macrophage phase. Following radiation therapy the degree of alveolitis, the pro-inflammatory phase of radiation-induced lung injury has been correlated with the degree of neutrophil influx to the treatment site [59]. Thus, in strains that respond with a lethal alveolitis, the lung infiltrate contains significantly more neutrophils and significantly fewer macrophages than strains that respond with fibrosing alveolitis [59]. Where cytotoxic cancer therapies result in cancer cell death via apoptosis, interaction, with apoptotic cells cause macrophages to secrete classic M2, anti-inflammatory cytokines, including IL-10 and TGF*β* [12, 60, 61]. Thus, it has been shown that exposure to dying cancer cells *in vitro* or in the tumor environment [26] activates the same innate immune programs as classical inflammatory resolution [62]. Both chemotherapy and radiation therapy have been shown to cause an influx of macrophages into the tumor [24–26, 63] and preventing macrophage influx [24, 25, 63], or preventing macrophages transitioning to an M2 resolution phenotype [26] increases the efficacy of therapy. In the process of radiation-induced cell death, cancer cells upregulate a range of receptors that can influence their phagocytosis and clearance (reviewed in [64]). Some molecules, such as calreticulin, have been proposed as potential pro-inflammatory factors in apoptotic cells. However, patients with higher levels of calreticulin expression in their tumor exhibit a worse prognosis than those with lower or absent expression [65]. This may relate to the coordinate regulation of CD47, which counteracts calreticulin and suppresses phagocytosis [65]. Thus, blockade of CD47 results in increased calreticulin-mediated uptake of dying cancer cells [65]. Combination of CD47 blockade with radiation therapy resulted in increased tumor control in murine models [66]. This dominant suppressive effect of CD47 *in vivo* may limit the potential positive effects of calreticulin. Conventional apoptotic stimuli upregulate calreticulin much more effectively than clinically relevant doses of radiation [67], but at extremely high *ex vivo* doses of 75 Gy where calreticulin is strongly upregulated, calreticulin is required for irradiated cells to act as a prophylactic vaccine [68]. Calreticulin appears to function as part of a group of proteins including CD91, C1q, and mannose binding lectin that serve to opsonize apoptotic cells for phagocytosis [69]. The uptake of these cells requires a signaling complex on phagocytes that includes integrin binding of MFG-E8 [70, 71] and Mer tyrosine kinase [72]. In addition, the C1q-apoptotic cell complexes can induce Mer expression in macrophages [73], which is associated with M2 macrophage differentiation [74] and results in immunosuppressive cytokine secretion by the phagocytic cells [74]. In the absence of MFG-E8 or Mer, mice develop autoimmune diseases associated with delayed clearance of apoptotic cells [70, 71, 75], and expression of Mer was critical for antigen-specific tolerance driven by apoptotic cells [76]. These data indicate that efficient phagocytosis and clearance of apoptotic cells normally functions to prevent immune activation in the absence of additional danger signals. However, manipulation of the myeloid response to dying cells has the potential to improve immune responses to tumor antigens *in vivo* [12, 77]. Similarly, since preventing the normal transition from inflammation to resolution interferes with wound healing, infectious agents [78] and immunological adjuvants reduce healing [79]. Importantly, infectious agents [80, 81] and immunological adjuvants [82, 83] have shown synergy with radiation therapy in the treatment of cancer. These data indicate that the functionality of the anti-tumor adaptive immune response following radiation therapy may be limited by inflammatory resolution at the tumor site, which directs repair of radiation-induced damage (Figure 1). Tumor macrophages are intimately involved in the transition to resolution and repair and represent an excellent target to manipulate the postradiation tumor environment.

This potentially positive role for tumor macrophages is highlighted by the fact that macrophages in the tumor environment can cross-present antigens from cancer cells following radiation therapy [84]. In this model, antigen presentation by the tumor macrophages following radiation was radiation dose dependent, transient, and was required for antigen-specific immune control of the tumor [84]. The antigen presentation function of macrophages in the tumor may be more limited by their inflammatory environment than by their cross-presentation capacity. Thus, while tumor macrophages can efficiently take up and present antigen, the presence of IL-10 (reviewed in [85]) and the absence of potent costimulatory molecules such as OX40L that are present in pro-inflammatory sites (reviewed in [86]) limit their ability to initiate immune responses locally [87, 88]. Dendritic cells have the intrinsic advantage of emigration, taking antigens out of the tumor for presentation in more permissive lymph nodes. In addition, lymph node macrophages present dead cell-associated antigens to T cells in the lymph node [89]. However, tumor-draining lymph nodes are also influenced by suppressive factors draining from the tumor environment, limiting their capacity to initiate tumor antigen-specific responses [90–92]. Thus, while the tumor environment is suboptimal for T cell stimulation activation by antigen presenting by myeloid cells, pro-inflammatory change in the tumor immune environment has the potential to dramatically change the capacity to stimulate T cells both in the tumor and in the tumor-draining lymph node.

## 3. Angiogenesis and Hypoxia

A hallmark of cancer is ongoing angiogenesis [7]. Continued production of angiogenic factors often results in over-abundance of new vascular sprouts but does not complete vessel development, resulting in poor vasculogenesis, inefficiencies in the blood supply and high interstitial pressure [93, 94]. These features limit penetration of drugs and macromolecules to the cancer cells despite the high permeability of tumor endothelium. In this way the poor fluid flow through tumors frequently results in a poor oxygen supply to cancer cells in tumors. Oxygen remains the single most relevant radiosensitizing agent, and clinical benefit is associated with reducing hypoxia in the tumor concomitant with radiation therapy [95]. Reducing the driving force of angiogenesis, for example, through VEGF inhibition, results in decreased interstitial pressure concomitant with fewer immature vessels or “vascular normalization” [94, 96]. In this way, antiangiogenic therapies have been shown to result in more mature vasculature, improved oxygen tension, and an improved response to radiation therapy [97–99].

While present in many parts of the tumor stroma, macrophages are known to accumulate in areas of tumor hypoxia [100] and are a critical driving force for angiogenesis in the tumor [101, 102]. Macrophages respond to hypoxia via HIF1a and upregulate VEGF in hypoxic regions of the tumor [103]. Deleting macrophage-derived VEGF has been shown to improve perfusion and decrease hypoxia in tumors, which interestingly resulted in increased tumorigenicity, demonstrating that cancer division is limited by the inefficiencies of the tumor stroma [104]. While the lower doses of fractionated radiation therapy can have biologic effects on the vascular endothelia, high-dose radiation therapy is known to kill endothelial cells [28]. Thus, following radiation there can be a transient decrease in angiogenic vasculature. However, directly or indirectly, radiation therapy causes an influx of myeloid cells including macrophages that are critical for endothelial regrowth following radiation therapy [63, 105].

These data suggest that macrophages contribute in two different ways to the vascular organization of the tumor and its interaction with radiation therapy. Firstly, as part of the tumor stroma, these cells respond to the hypoxia caused by cancer cell growth and division to generate new blood vessels through upregulation of VEGF. This constant pressure of growth and hypoxia creates a constant state of neoangiogenesis, without converting to fully functional vasculature. The result is high interstitial pressure, poor fluid penetration, and patchy hypoxia. These are features of the “wound that does not heal,” and it is this environment that harbors cancer cells at low oxygen tension in a radiation-resistant state. Secondly, following radiation induced damage to the tumor, macrophages are recruited to the tumor as part of the repair process. In this phase macrophages and their repair of the tumor environment permit outgrowth of residual cancer cells. It is possible that targeting these two phases requires different approaches. For example, in mouse models antiangiogenic therapy was most effective when given in a narrow window a few days in advance of radiation therapy, to optimally increase the oxygen tension at the time of treatment [97–99]. Following treatment, it may be more effective to target the macrophage influx directly [25, 63, 105], since in addition to their VEGF-mediated vascular effects, these cells are involved in multiple other elements of the tumor response to radiation.

While VEGF is an effector cytokine, it may also be a marker of the “wound healing” phenotype of the tumor, and thus the responsiveness to treatment. Studies in colorectal cancer patients have shown that infiltration of CD8 T cells and expression of VEGF represent opposing predictors of recurrence [106]. These markers were most effective in combination; patients whose tumors exhibited high VEGF had a poor prognosis regardless of cytotoxic CD8 infiltrate; however, those patients with low VEGF and high cytotoxic CD8 infiltrate displayed an excellent prognosis [106]. These data suggest that VEGF expression is a marker of immunosuppressive inflammatory resolution in tumors, which is dominant even in the presence of a strong cytotoxic CD8 T cell infiltrate. Macrophages in hypoxic conditions upregulate a number of M2-associated immune suppressive genes, including IL-10 and arginase (reviewed in [107]). Interestingly, in murine models, deleting HIF1a in macrophages was shown to improve T cell function and tumor control, though surprisingly HIF1a deletion had this effect without influencing the vascularity of the tumor [108]. These data suggest that the macrophage response to hypoxia has additional effects beyond the vasculature, and that there may be a close interplay between adaptive and innate immune cells in the tumor response to radiation therapy.

## 4. MDSC, Granulocytes, and Macrophages

While macrophages have been the myeloid population most studied in tumor biology, recently there has been a strong interest in the newly defined population called myeloid-derived suppressor cells (MDSCs) [109]. This encompasses both a functional definition, in suppression of T cells *in vitro*, and a phenotypic definition, which initially focused on expression of Gr1 in murine models. Certain murine models of cancer are associated with dramatic myeloid expansions, resulting in gross splenomegaly and log expansions of myeloid cells in the peripheral blood. Spontaneous murine tumor models also display myeloid expansions; these have been described in transgenic models of mammary and pancreatic carcinomas [110–112]. In patients, myeloid expansions have been described in a range of cancers [113–117], though they do not reach the extent seen in some of the more aggressive murine models. The Gr1 phenotypic marker commonly used in murine models does not translate to human myeloid cells. However, in both the murine and human examples, the MDSC designation encompasses classically defined neutrophils and monocytes. The Gr1 antibody binds both Ly6G and Ly6C, and when these markers are used together, it is possible to distinguish Ly6C^+^Ly6G^−^ monocytic cells from Ly6G^+^ neutrophils. While both populations are expanded in addition to Ly6C^−^Ly6G^−^ (Gr1^−^) monocytes, in the peripheral blood, the Ly6C^+^Ly6G^−^ monocytic cells exhibit greater suppressive activity than the Ly6G^+^ neutrophils [118, 119]. In patients, the granulocytic population can be distinguished from monocytes by size and granularity more conveniently than in mice, as well as the granulocyte marker CD15 and the monocyte marker CD14. Similarly to murine models, suppressive activity is found in monocytic cells, particularly in a subpopulation of CD14^+^ monocytes characterized by low expression of HLADR [117]. Expansion in these HLADR^lo^CD14^+^ monocytes has been correlated with invasive disease in cancer patients [113, 114, 120].

Myeloid expansion from progenitors and their initial differentiation into the variety of myeloid subpopulations including monocytes and granulocytes are dependent on the relative levels of the growth factors M-CSF, GM-CSF, and G-CSF. These act on the available pool of progenitors and cross-compete; thus, M-CSF deficient mice have absent monocytes but increased numbers of granulocytes [121]. Engineering cancer cells to stably expressed GM-CSF or exogenous addition of GM-CSF was shown to result in expansion of Gr1^+^ myeloid cells [122]. By contrast, antibody inhibition of GM-CSF results in a some decrease in CD11b^+^Gr1^+^ cells in tumor-bearing animals [118], and the presence of GM-CSF has been strongly associated with the myeloid expansion in spontaneous pancreatic cancer models [123]. G-CSF is associated with the extreme myeloid expansions of specific models [124, 125], and in these models, antibody inhibition of G-CSF and not GM-CSF or M-CSF reversed accumulation of Ly6G^+^ cells in tumors and lung metastases [124]. Since G-CSF is required for neutrophil differentiation, this growth factor may be responsible for the less suppressive but more dramatic granulocyte expansion characteristic of the murine models [126]. Interestingly, exogenous administration of either GM-CSF or G-CSF to animals can provide some protection against lethal radiation doses [127–129] and has been delivered to patients following radiation accidents [130]. In addition to hematopoietic recovery, the effect of these growth factors may relate to neutrophil migration to irradiated sites and subsequently improved repair of radiation damage [131]. These data fit with the repair role of myeloid cells in the tumor discussed earlier, and in this context of cancer-driven myeloid expansion, GM-CSF and G-CSF may improve recovery of the tumor from radiation damage, permitting outgrowth of residual cancer cells.

Treatment of murine tumors with chemotherapy [132–134] and surgical resection [132, 135] has been shown to limit or reverse the myeloid expansion. We recently demonstrated that radiation therapy of murine tumors also reversed the systemic Gr1^+^ myeloid expansion associated with tumor growth (Crittenden et al., submitted). In common with surgical and chemotherapy, untreated metastatic disease and residual disease at the treatment site prevented a full normalization of myeloid numbers following radiation therapy, and tumor recurrence resulted in a renewed myeloid expansion (Crittenden et al., submitted). These data suggest a close link between tumor burden and myeloid expansion, and that tumor treatment with radiation therapy, chemotherapy, or surgical excision can transiently improve the systemic immune environment by reducing the number of immune suppressive myeloid cells. 

## 5. The Cross-Regulation between Polarized Macrophages and T Cells

Recent data demonstrates that T cells can play an important role in the efficacy of radiation therapy [17, 18], and the interplay between T cells and radiation therapy has been summarized in a number of recent reviews [16, 30, 136]. Once T cells are recruited to the tumor environment, tumors combine a poor environment for T cell activation and a high expression of factors that suppress adaptive immunity. For example, cancer cells, as well as stromal cells in the tumor environment, are abundant sources of TGF*β* [137]. Exposure to TGF*β* has been shown to divert macrophage pro-inflammatory responses towards M2 macrophage responses, characteristic of inflammatory resolution [138, 139]. These resolution macrophages then become an additional source of TGF*β* [61]. As we have discussed, TGF*β* is a critical cytokine in effective wound healing, and the regulation of wound healing responses requires interplay between adaptive immune cells, macrophage differentiation, and the regulation of TGF*β* expression. Systemic administration of TGF*β* accelerates wound healing [140, 141], while administration of IFN*γ*, which directs M1 differentiation of macrophages, results in delayed wound healing in mice [142]. Similarly, administration of IFN*γ* reduced fibrosis in rat models of hepatic fibrosis [143], and long-term administration of IFN*γ* reduced fibrosis in patients with chronic Hepatitis B infections [144]. In mice lacking IFN*γ*, there is an enhanced induction of TGF*β* following injury that results in accelerated wound healing [145]; together these data suggest that the adaptive immune response works against wound healing. TGF*β* is also an effective immune suppressive cytokine; blockade of tumor-derived TGF*β* renders tumors sensitive to adaptive immunity [146], and T cells rendered unresponsive to TGF*β* are more effective in tumor control [147]. TGF*β* can potently direct CD4 T cells to differentiate into T regulatory cells, defined by expression of FoxP3 and suppressive activity on naïve T cell proliferation [148]. These T regulatory cells are found at increased levels in tumors, with a high level of T regulatory cell infiltration correlated with poor prognosis [149, 150]. Therapeutic depletion of T regulatory cells can significantly increase anti-tumor immune responses [151, 152]. As discussed earlier, TGF*β* is expressed in the target site at later time points following radiation therapy [49, 50]. Thus, in its role as an inflammatory resolution cytokine, radiation-mediated TGF*β* induction may also cut off T cell effector function at the tumor site to permit wound repair. Recent studies demonstrate that TGF*β* inhibitors are able to block the toxicity associated with radiation-mediated fibrosis [153], increase the therapeutic efficacy of radiation therapy in murine models [154, 155], and synergize with immunotherapy [156]. These data suggest that the key regulators of inflammatory resolution are potential targets to increase the efficacy of radiation therapy.

TGF*β* is not the sole source of adaptive immune suppression in inflammatory resolution. A characteristic feature of M1 and M2 macrophage differentiation is their method of L-arginine metabolism [157]. M2 macrophages characteristically express the enzyme arginase I while M1 macrophages characteristically express iNOS. While L-arginine breakdown products may have direct effects on other cells, the consumption of L-arginine by macrophages expressing high levels of arginase can suppress T cell activation *in vitro* and *in vivo* [158, 159]. L-arginine deficiency may be an explanation for the low expression of CD3 zeta chain that is frequently observed in tumor-infiltrating T cells [160, 161], which results in a relative unresponsiveness to TCR stimulation that is reversible on *ex vivo* culture [162]. Arginase I activity is induced in myeloid cells following trauma [163] including trauma as a result of surgery [164] and correlates with increased detectable IL-10, supporting evidence for M2 polarization and initiation of inflammatory resolution and wound healing responses. In models of infectious disease, macrophage polarization and induction of arginase can play a key role in regulating pathology, but also in persistent infection. In Schistosoma infection, induction of a Th2 and thus M2 immune response driven by IL-4 and IL-13 reduces the acute toxicity of infection but results in chronic disease [165, 166]. In non-healing leishmaniasis lesions, M2 differentiation and expression of arginase results in local suppression of effector T cell responses; inhibition of arginase or exogenous provision of L-arginine resulted in increased numbers of effector cells, decreased numbers of infectious agents, and decreased lesion size [167]. These data demonstrate that tumor-driven M2 differentiation of macrophages and induction of arginase gene expression are very similar in mechanism and outcome to both wound healing and inflammatory resolution following infection. The precise role of the arginase enzyme is puzzling, as it remains unclear whether L-arginine availability, suppressive arginase metabolites, or removal of L-arginine as an iNOS substrate is the major role of arginase in wound healing [157]. While it is tempting as an immunologist to see arginase primarily as an immunosuppressive molecule, the differentiation program resulting in arginase expression may be intended to provide sources of free proline for the synthesis of collagen in wound healing [168]. Nevertheless, the regulation of macrophage arginase activity demonstrates the dual role of wound repair and adaptive immune suppression in effective inflammatory resolution.

As described earlier, M2 macrophages secrete a number of anti-inflammatory cytokines in addition to TGF*β*, including IL-10 [12, 60, 61]. IL-10 is an important immunoregulatory cytokine; mice deficient in IL-10 display abnormally prolonged inflammatory responses [169], and infectious agents exploit the immunoregulatory actions of IL-10 to extend infection in the host [170]. The mechanisms of IL-10 mediated immune suppression occur through a combination of targets. One critical target is antigen presenting cells, which respond to IL-10 by downregulating antigen processing and presentation [171, 172], and IL-10 have been shown to feedback on macrophages to increase alternative macrophage differentiation [173]. T cells are another major target for the action of IL-10, which selectively suppresses the Th1-type adaptive immune responses that are particularly desired for immune control of tumors. Thus, the progressive induction of IL-10 in tumor infiltrating cells during tumor growth has been shown to suppress anti-tumor adaptive immune responses [174]. IL-10 can be effectively blocked with specific antibodies to the cytokine and to the IL-10 receptor, and each have been shown to enhance the adaptive immune response [87] resulting in more effective immune control of tumors [175]. Thus, the published data shows that M2 macrophage production of IL-10 is a key effector element of inflammatory resolution, and the absence of IL-10 results in a prolongation of inflammatory destruction and adaptive immunity. 

With the FDA approval of CLTA-4 blocking antibodies and the clinical development of other T cell “checkpoint” targets including PD1, multiple strategies will be open to prolong T cell activation in suppressive environments. Nevertheless, the dominant pressure for resolution faced by all immune responses can result in adaptive immune shutdown through alternative means. Thus, when given anti-CTLA-4 blocking antibodies the negative regulation mediated by CTLA4 is blocked, TGF*β* and IL-10 remain in the postradiation tumor environment, and it is likely that these factors will continue to limit adaptive immune function despite CTLA4 blockade. It may not be feasible to systemically target each of these pathways without toxicity. For example, blocking CTLA4 activity through the drug Ipilimumab frequently results in colitis or other assorted inflammatory disorders as a result of deregulated adaptive immune activity [176]. Similarly, IL-10 knockout mice develop chronic colitis as a result of deregulated T cell immunity [169], while TGF*β* knockout mice can develop multifocal inflammatory disease [177]. However, since radiation therapy causes a transient inflammation before resolution occurs, radiation treatment may provide an opportunity to focally “reset” the immune environment in the treated tumor. In this way, preventing the onset of resolution may only have effects in the treatment field.

## 6. Implications for Radiation Therapy and Immunotherapy

These discussions demonstrate that immune biology is not fixed. Radiation therapy causes a flux in the immune biology of the tumor, and in the regulation of this flux are opportunities to extend radiation-mediated damage to the tumor and improve clearance of residual cancer cells and prime immune responses to target distant disease. In view of the central role for M2 differentiation of macrophages in resolution and repair, we propose that strategies that prevent M2 differentiation of tumor macrophages can be key to extending inflammatory destruction and adaptive immune responses in the tumor [2]. Of course, such effects are not always desired. For example, in clinical scenarios such as postexcision radiation of a tumor bed, this could cause unacceptable toxicity to what is mostly normal tissue. However, where a tumor located in a tissue that can withstand inflammatory destruction is treated upfront with radiation therapy, manipulating the inflammatory state of the tumor may be safe, feasible, and beneficial. While the data we have presented makes a strong case that inflammatory resolution and tissue repair limit adaptive immunity in the tumor following radiation therapy and permit tumor recurrence, this may not be very relevant if there is not a strong anti-tumor immune response in the first place. In patients with poor reactivity to tumor antigens, immunotherapies that efficiently initiate new anti-tumor immune responses may be a necessary part of effective radiation therapy.

Since inflammatory resolution and repair can be prevented by adaptive immune cytokines [142–144], and since strong adaptive immune responses can remodel the tumor environment [178], it is possible that a sufficiently potent adaptive immune response can hold off wound healing, prevent cancer outgrowth, and complete regression following radiation therapy. (Figure 1). In preclinical models of radiation therapy combined with immunotherapy, tumor-antigen-specific T cells engineered to overexpress IL-12 were able to direct tumor regression where unmodified T cells were not [179]. The mechanism of IL-12 action included removing myeloid suppression of T cells in the tumor environment [180]. Agonistic antibodies to OX40 have been shown to increase T cell influx into the tumor environment, decrease macrophage suppression [181], and synergize with radiation therapy to cure tumors in mice [18]. Blocking antibodies to CTLA-4 cause T cell infiltration into tumors in mice [182] and patients [183], CTLA-4 blockade synergizes with radiation therapy in murine models [184], and the combination has been associated with a case of tumor regression in patients [185]. This ability of immunotherapy to direct T cells to the tumor site and through cytokines change the tumor immune environment may be critical for their ability to synergize with radiation therapy. High dose IL-2, which is well known to cause lymphocyte egress from the peripheral blood, has been shown to cause lymphocytes to accumulate at tumor sites [186], and in a recent study from our institution, investigators demonstrated durable cures of widely metastatic disease when high dose IL-2 immunotherapy was combined with radiation therapy in a phase I clinical study [187]. High dose IL-2, with its accompanying toxicities, is possibly an extreme example of what immunotherapy can do, but we propose that to generate cures without addressing the suppressive force of macrophage-driven inflammatory resolution, immunotherapy will require extremely strong immune stimuli. This tradeoff between toxicity and efficacy is a familiar one to both radiation oncologists and immunologists. However, since inflammatory resolution caused by tumor macrophages can limit the efficacy of immunotherapies even in the absence of radiation therapy [188], it is likely that targeting macrophage-driven inflammatory resolution will be a valuable addition to many existing immunotherapy approaches.
